# Drug Induced Liver Injury: Can Biomarkers Assist RUCAM in Causality Assessment?

**DOI:** 10.3390/ijms18040803

**Published:** 2017-04-11

**Authors:** Rolf Teschke, Johannes Schulze, Axel Eickhoff, Gaby Danan

**Affiliations:** 1 Department of Internal Medicine II, Division of Gastroenterology and Hepatology, Klinikum Hanau, D-63450 Hanau, Germany; Axel_Eickhoff@klinikum-hanau.de; 2 Teaching Hospital of the Medical Faculty, Goethe University Frankfurt, D-60590 Frankfurt, Germany; 3 Institute of Occupational, Environmental and Social Medicine, Medical Faculty, Goethe University Frankfurt, D-60590 Frankfurt, Germany; j.schulze@em.uni-frankfurt.de; 4 Pharmacovigilance Consultancy, F-75020 Paris, France; gaby.danan@gmail.com

**Keywords:** drug induced liver injury, DILI, RUCAM, Roussel Uclaf Causality Assessment Method, diagnostic biomarkers, pathogenesis

## Abstract

Drug induced liver injury (DILI) is a potentially serious adverse reaction in a few susceptible individuals under therapy by various drugs. Health care professionals facing DILI are confronted with a wealth of drug-unrelated liver diseases with high incidence and prevalence rates, which can confound the DILI diagnosis. Searching for alternative causes is a key element of RUCAM (Roussel Uclaf Causality Assessment Method) to assess rigorously causality in suspected DILI cases. Diagnostic biomarkers as blood tests would be a great help to clinicians, regulators, and pharmaceutical industry would be more comfortable if, in addition to RUCAM, causality of DILI can be confirmed. High specificity and sensitivity are required for any diagnostic biomarker. Although some risk factors are available to evaluate liver safety of drugs in patients, no valid diagnostic or prognostic biomarker exists currently for idiosyncratic DILI when a liver injury occurred. Identifying a biomarker in idiosyncratic DILI requires detailed knowledge of cellular and biochemical disturbances leading to apoptosis or cell necrosis and causing leakage of specific products in blood. As idiosyncratic DILI is typically a human disease and hardly reproducible in animals, pathogenetic events and resulting possible biomarkers remain largely undisclosed. Potential new diagnostic biomarkers should be evaluated in patients with DILI and RUCAM-based established causality. In conclusion, causality assessment in cases of suspected idiosyncratic DILI is still best achieved using RUCAM since specific biomarkers as diagnostic blood tests that could enhance RUCAM results are not yet available.

## 1. Introduction

Drug induced liver injury (DILI) commonly refers to idiosyncratic DILI and remains a diagnosis of exclusion [1,2,3,4,5,6,7,8,9,10,11]. This requires a clear differentiation from other liver diseases [1,3], which may confound the DILI diagnosis [8,9,10,11] and prevail with high incidence and prevalence rates in many countries [12,13,14]. Among the most common diseases to be excluded are chronic liver diseases such as alcoholic liver disease (ALD), non-alcoholic fatty liver disease (NAFLD) and its complication non alcoholic steatohepatitis (NASH), and acute or chronic viral infections, namely by hepatitis A virus (HAV), hepatitis B virus ((HBV), hepatitis C virus (HCV), and hepatitis E virus (HEV), as evidenced by specific serum biomarkers [3,8,9,12]. Compared to chronic liver diseases [12,13,14], prevalence and incidence rates of DILI are much lower, around 14/100,000 inhabitants [15,16,17,18]. In patients with abnormal liver tests (LTs), the diagnosis will be therefore much more likely a chronic liver disease than a DILI.

The exclusion of alternative causes in cases of suspected idiosyncratic DILI is one of the key elements of RUCAM (Roussel Uclaf Causality Assessment Method), the preferred diagnostic approach to assess causality in such setting [1,2,8,9,10,11]. Other tools to identify DILI focus on biomarkers as possible diagnostic tests. However, their performance indicators in idiosyncratic DILI are not yet settled since definitions of DILI and biomarkers are variable and vague. For instance, some reports defined idiosyncratic DILI as liver injury caused by synthetic drugs, while other publications included also single herbs, products of herbal mixtures, and dietary supplements as suspected agents. Additional reports considered cases of intrinsic DILI due to acetaminophen overdose or intrinsic herb induced liver injury (HILI) caused by hepatotoxins like unsaturated pyrrolizidine alkaloids (PAs). Finally, blood biomarkers as aids for clinicians are under evaluation for various purposes like early detection of liver injury, assessment of disease severity, prediction of prognosis, or strong diagnostic tool in a routine clinical setting.

In this review, article, newly proposed biomarkers with their advantages and limitations of their potential use for DILI case assessment are discussed. The focus will be on whether these biomarkers could assist the RUCAM-based evaluation of suspected idiosyncratic DILI. Such biomarkers would represent a major progress in the difficult diagnosis of a complex disease.

## 2. Methods

Relevant studies and reports were identified using a computerized search of the Medline database that included publications from 1993 to 15 February 2017. We used the following terms: RUCAM, causality assessment, biomarker, drug hepatotoxicity, and drug induced liver injury. For each term, we considered the first 50 publications representing specific reports or review articles. The reference lists of the retrieved publications were hand searched for additional publications. Subsequently, we reviewed selected reports relevant to the aim of our study and focused on publications in English language.

## 3. Definitions

Biomarkers of a liver injury remain an interesting topic in clinical hepatology and are the subject of abundant publications, some of these are specifically referenced [1,19,20,21,22,23,24,25,26,27,28,29,30,31,32,33,34,35,36,37,38,39,40]. DILI and biomarkers are so complex that any proposals are controversial and uncertainties remain due to insufficient definitions of the two entities.

### 3.1. Biomarkers

In the literature search using the term “biomarker definition in drug induced liver injury“, the report of the original RUCAM of 1993 [1], quoted by 1050 publications, was presented on top, namely place one, of a long list of publications that provided overall 1,310,000 hits for the search terms used. According to the definition proposed in 1993, a biomarker is a laboratory test sensitive and specific enough to confirm the drug-related nature of a liver injury [1]. This definition is still valid and was expanded in recent years. Indeed, biomarkers are single or a panel of proteins, nucleic acids, or protein-metabolite adducts. If the described biomarkers are drug independent and merely assess liver injury due to any cause, the term “DILI biomarker” is incorrect and should not be used. Ideally, a biomarker of DILI must not only be the signature of a liver injury but also identify the offending drug or, at least, a class of chemical entities. Most biomarkers under investigation are of limited value because they are based on animal studies, lack of validation in humans, and are not easily available in clinical practice.

### 3.2. Liver Injury

**Figure 1:**
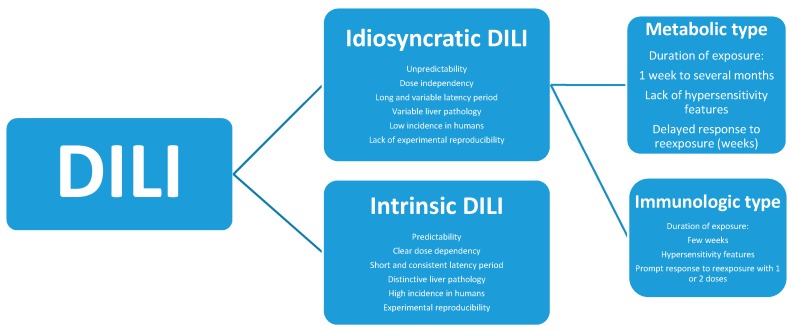
Characteristics of idiosyncratic DILI and intrinsic DILI. Adapted from a previous report [10]. Abbreviation: DILI, Drug induced liver injury.

DILI is classified according to the idiosyncratic or intrinsic toxicity of the offending product (Figure 1). Specific criteria differentiate idiosyncratic DILI from intrinsic DILI. In addition, DILI refers not only to drugs but also to herbs, herbal mixtures, herbal products, and dietary supplements. Liver injury due to non-drug products should better be classified as herb-induced liver injury (HILI) [40,41,42] with the same DILI classification: idiosyncratic HILI and intrinsic HILI.

## 4. Key Issues of Biomarkers in Suspected DILI

Back in 1990s, there was no consensus on a laboratory test sensitive and specific enough to confirm the drug-related nature of an idiosyncratic liver injury. The lack of a validated biomarker and other considerations such as the disputed subjective opinion of experts assessing individual DILI cases led to set up RUCAM [1]. This CAM (Causality Assessment Method) was validated by using cases of liver injury with positive rechallenge [2] and is applied worldwide to help assess causality for suspected DILI and HILI [3]. Establishing a new and valid biomarker for idiosyncratic DILI requires DILI series with verified diagnosis and robust causality assessment. This should be achieved by using RUCAM [3], putting aside vague opinion-based and unstructured approaches [3,10].

Yet in 2016, the EMA (European Medicines Agency), in the frame of IMI (Innovative Medicines Initiative) projects and more precisely the SAFE-T (Safer and Faster Evidence-based Translation) consortium [19], published a Letter for support of DILI biomarker [20] where the lack of sensitive and specific clinical tests to diagnose, predict and monitor idiosyncratic DILI are addressed again and considered these limited tools as a major hurdle in drug development when it comes to evaluate a liver injury. The EMA letter seems to be preliminary, with no straight forward proposals in clinical trials and clinical practice [20]. The need of DILI biomarkers in clinical trials is described by the EMA as follows [20]: (1) early or earlier detection of DILI as compared to current diagnostic rules; (2) ability to predict DILI outcome, with particular emphasis on severe DILI and acute liver failure; (3) prognosis and monitoring of progression and regression of DILI; (4) assessment of liver adaptation; and (5) searching for and predicting of early intrinsic liver injury in clinical trials [20]. Several described biomarkers received critical comments [21], as summarized in Table 1.

Two other reports analyzed the potential value of new biomarkers in DILI [21,22]. They detail several considerations with respect to their validity [21,22]. From different viewpoints, all three publications addressed issues of biomarkers to improve the diagnosis of DILI [20,21,22], while other reports provided additional information [23,24,25,26,27,28,29,30,31,32,33,34,35,36,37,38,39,40].

### 4.1. Early Detection

The EMA letter asked for one or a set of biomarkers for an early diagnosis of DILI as compared to current diagnostic rules (Table 1) [20]. Among the potential biomarkers there were CK-18 (Cytokeratin-18), microRNA-122 (microarray RNA-122), total HMGB-1 (High Mobility Group Box protein-1), GLDH (Glutamate dehydrogenase), SDH (Sorbitol dehydrogenase) proposed as marker for hepatocyte necrosis, ccCK-18 (caspase-cleaved CytoKeratin-18) proposed as marker for apoptosis, hyperacetylated HMGB-1, and MCSFR-1 (Macrophage colony-stimulating factor receptor-1) proposed as marker for immune activation (Table 1) [20]. Other proposals included M-30 (apoptosis), M-65 (apoptosis/necrosis), and microRNA-192 (unspecified liver damage) [21]. Some of the proposed biomarkers are not liver or not drug specific, others are difficult to be assessed due to the requirement of mass spectroscopy (Table 1) [21]. Microarray RNAs (microRNAs) including microRNA-122 have been evaluated in experimental liver injury and in human intrinsic DILI caused by *N*-acetyl-para-aminophenol (APAP), also called acetaminophen or paracetamol, but uncertainty exists on their diagnostic value in human idiosyncratic DILI [40]. Microarray RNAs circulating in the blood may be found with low levels in healthy individuals and with higher levels in patients with various diseases such as cancer, cell and organ transplantation, coronary heart disease, stroke, sepsis, burns, or confined to specific organs [27,40]. Of more interest and better studied are the mechanisms by which microRNAs leave damaged cells and enter the blood [40].

For many decades, serial analyses of routine LTs (liver tests) like ALT (alanine aminotransferase), AST (aspartate aminotransferase), ALP (alkaline phosphatase), and total bilirubin (TBIL) have traditionally been used for detection of DILI with marketed products and during clinical trials [1,2,3]. In trials, however, a more stringent diagnostic approach is suitable in order to ensure the safety of subjects by detecting incipient DILI that may otherwise be missed using traditional LTs (Table 1) [20,21]. To fill up this gap, various new biomarkers are under consideration that hopefully will have a specificity and sensitivity high enough to out-perform and replace in the future traditional LTs [20,21,22,23,24,25,26,27,28,29,30,31,32,33,34,35,36,37,38,39,40]. However, it seems that none of the biomarkers listed under early recognition of liver injury fulfills these requirements (Table 1) [20,21]. In each case of suspected liver injury, additional approaches are required like RUCAM to identify this reaction as drug-related event after exclusion of alternative causes [3] that are much more frequent than DILI [9]. Neglecting hepatic co-morbidities including flares of a pre-existing chronic liver disease [43,44] or viral infections such as HEV [45] can lead to an erroneous DILI diagnosis, even in a clinical trial setting [45].

**Figure 2:**
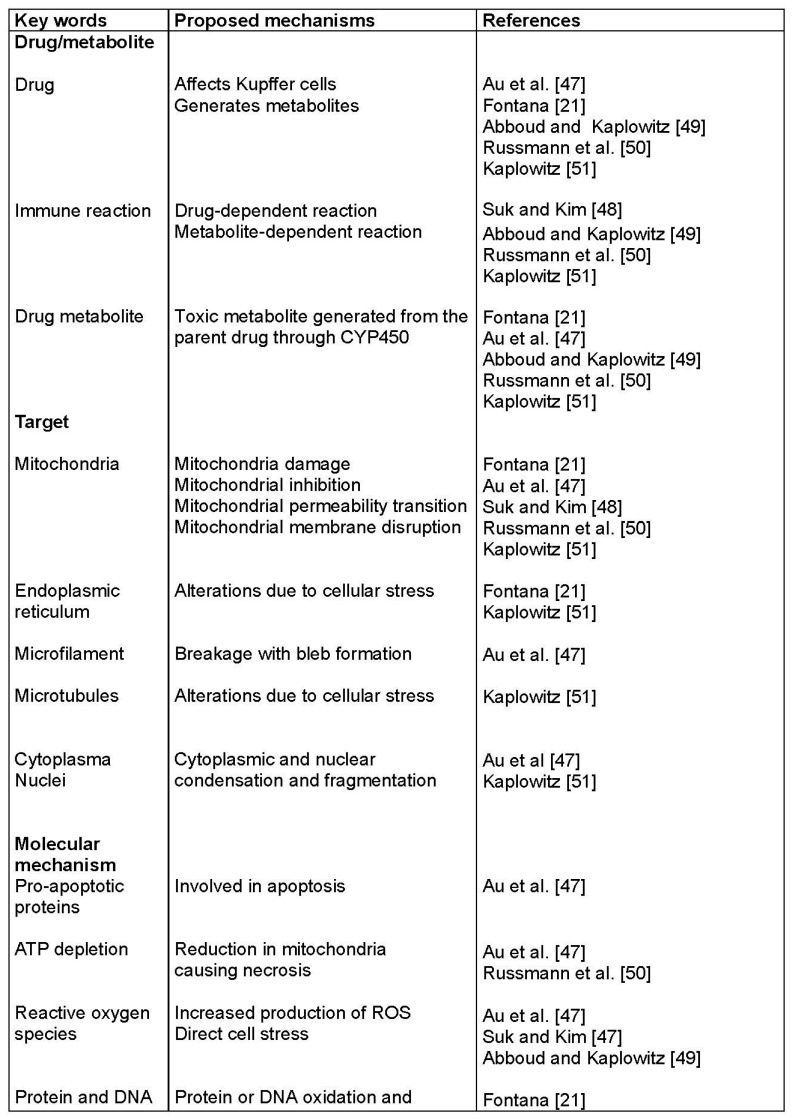

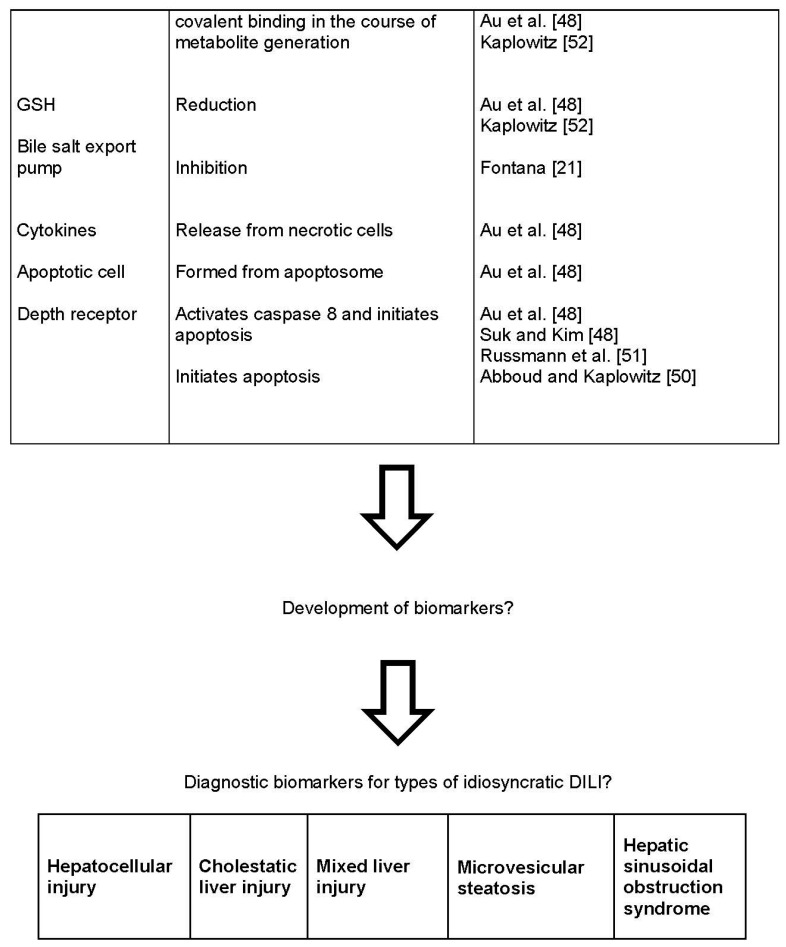
Selected pathogenetic mechanisms proposed for idiosyncratic DILI. Abbreviations: CYP450, Cytochrome 450; DILI, Drug induced liver injury; GSH, Glutathione; ROS, Reactive oxygen species.

In clinical practice with marketed products, early recognition of DILI by prospective use of specific biomarkers is hardly possible since the patients usually do not consult at the early phase of the liver injury. Instead, ALT or ALP should be used as recommended for DILI evaluation in connection with causality assessment using RUCAM [1,2,3,23]. The exclusion of alternative causes according to the clinical setting and a predefined list is mandatory to reduce the risk of wrong DILI diagnosis (Figure 2) [3]. At the moment, the use of traditional low cost LTs is the most convenient approach. They are available everywhere and allow for an early suspicion of DILI without the need of a battery of other, costly laboratory tests including possible biomarkers of unknown sensitivity and specificity (Table 1) [21,22]. They should be performed as soon as clinical symptoms appear including abdominal discomfort, nausea, and reduced appetite as early warning signs, while jaundice, pruritus, dark urine and pale stools are late and indicate dangerous stages of progressing liver injury [43].

**Figure 3:**
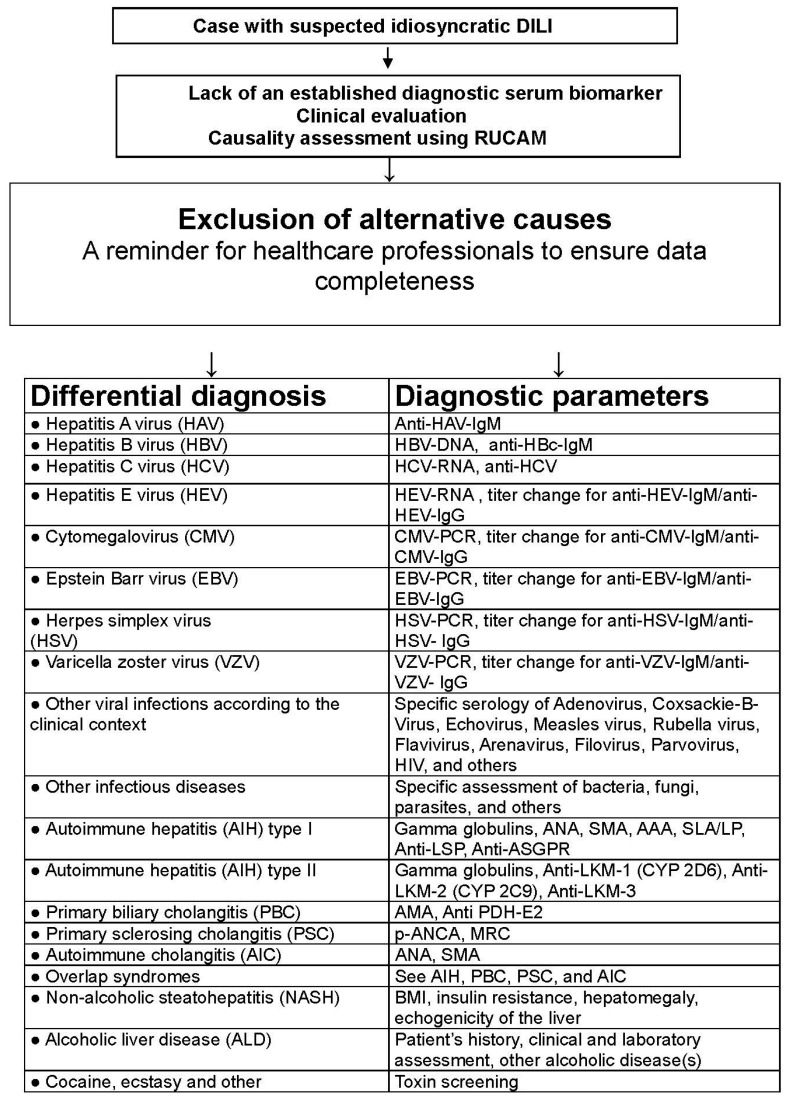

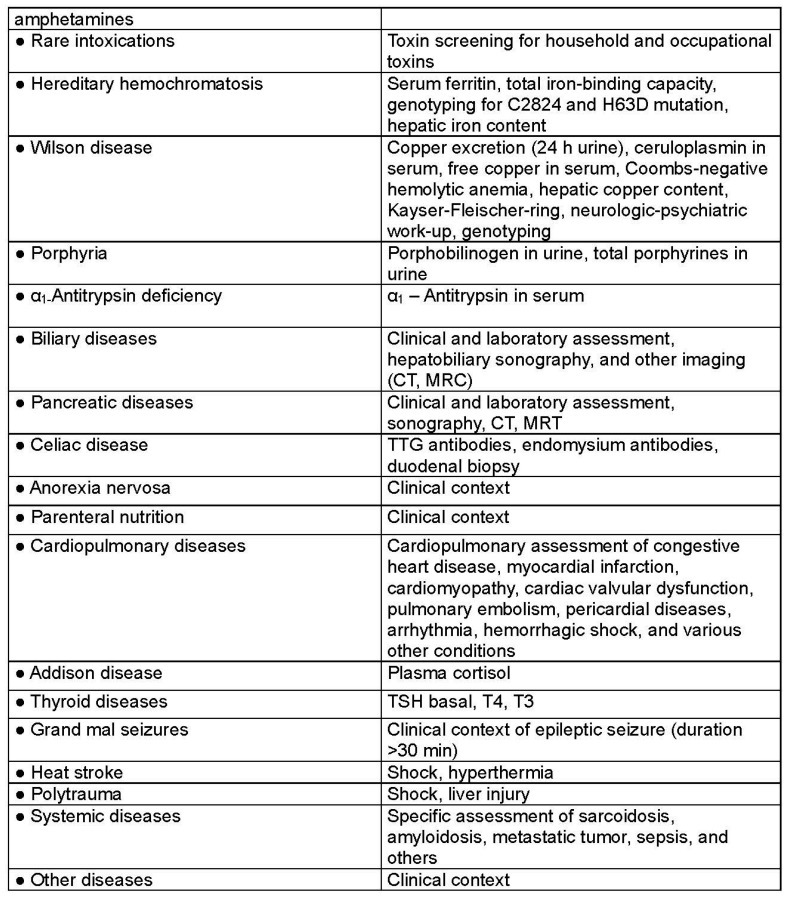
Checklist of differential diagnoses in cases of suspected idiosyncratic DILI. This tabular listing is adapted and derived from a previous publication [3]. Although not comprehensive, it is to be used as a guide and in connection with RUCAM [3]. Abbreviations: AAA, Anti-actin antibodies; AMA, Antimitochondrial antibodies; ANA, Antinuclear antibodies; ASGPR, Asialo-glycoprotein-receptor; BMI, Body mass index; CT, Computed tomography; CYP, Cytochrome P450; DILI, Drug induced liver injury; DPH, Pyruvate dehydrogenase; HAV, Hepatitis A virus; HBc, Hepatitis B core; HBV, Hepatitis B virus; HCV, Hepatitis C virus; HEV, Hepatitis E virus; HIV; human immunodeficiency virus; LKM, Liver kidney microsomes; LP, Liver-pancreas antigen; LSP, Liver specific protein; MRC, Magnetic resonance cholangiography; MRT, Magnetic resonance tomography; p-ANCA, Perinuclear antineutrophil cytoplasmatic antibodies; PCR, Polymerase chain reaction; RUCAM, Roussel Uclaf Causality Assessment Method; SLA, Soluble liver antigen; SMA, Smooth muscle antibodies; TSH, Thyroid stimulating hormone; TTG, Tissue transglutaminase.

Based on DILI and HILI cases with a high causality grading assessed by RUCAM [3], evidence is still lacking to validate new sensitive and specific diagnostic biomarkers that would allow early recognition of true idiosyncratic drug-induced liver injury (Table 1, Figure 3) [20,21,22,23].

### 4.2. Prediction of Outcome

Valid biomarker(s) that would predict DILI outcome, with particular emphasis on severe DILI and acute liver failure [20] are not yet identified, nor was any procedural approach discussed (Table 1).

### 4.3. Prognosis and Progression

Assessing DILI prognosis and monitoring disease progression are important issues related to disease severity [20]. Proposals included hyperacetylated HMGB-1, Osteopontin, total Keratin-18 and MCSFR-1 (Table 1), which had been selected for their possible potential as clinical DILI biomarkers, should be incorporated into future clinical trials to anticipate a risk for progression of hepatocellular injury to severe DILI [20]. For this investigational study, it is proposed that patients with an initial DILI diagnosis based on elevations of ALT alone or in combination with TBIL should be included [20]. However, concern has been expressed that HMGB-1 may not be liver specific (Table 1) [21].

Currently, in clinical trials as well as clinical practice, disease severity of suspected DILI is well assessed by ALT and TBIL [1,2,3,23] in order to detect an hepatocellular injury and jaundice that would impose to assess causality and if it is a probable DILI, to discontinue the drug treatment [43,44,46]. This recommendation known as Hy’s law after the late Hyman Zimmerman, is based on a 10% mortality risk of DILI if the following three criteria are met: (1) serum ALT or AST >3 × ULN, combined with serum TBIL elevated to >2 × ULN; (2) exclusion of a cholestatic component (the liver injury should be of hepatocellular type, ratio R should be >5) as outlined in the RUCAM description [3]; and (3) no other cause of acute or chronic liver injury can be found. The latter condition requires a careful exclusion of alternative causes (Figure 2), which is a RUCAM component [3]. Such serum ALT and bilirubin values may be indicators for ongoing severe liver injury and/or biomarkers for severe liver injury [43,44,46]. It is unclear whether other parameters or new biomarkers perform better (Table 1) [21,22,23].

Expectations were high with the use of a software program named eDISH (evaluation of Drug-Induced Serious Hepatotoxicity), which was established in order to facilitate early recognition of a safety signal in clinical trials, partially based on Hy’s law [47]. However, eDISH is not applicable for a single case of suspected DILI as it was established for clinical trials. In addition, suspected severe DILI detected by eDISH should be validated only after causality assessment by a robust method such as RUCAM [3] resulting in a probable or highly probable causality grading [43].

### 4.4. Regression

Monitoring disease regression was considered another important point deserving one or a set of new biomarkers, but proposals were not provided how this issue should be handled (Table 1) [20]. RUCAM clearly described details how serum ALT or ALP decrease following drug cessation [1,3].

### 4.5. Adaptation

Biomarkers are also needed to predict liver adaptation. In other words, to identify patients who will worsen DILI from those who will recover from the initial injury despite drug treatment continuation (adaptors) (Table 1) [20]. However, the rationale for the need of a new biomarker is unclear, since ALT, AST, ALP or TBILI are already available to monitor the liver adaptation, although they cannot predict such process [43,48]. Indeed, liver adaptation or tolerance with small LT increases [43,48] below the threshold values of liver injury [3] is commonly observed during treatment with drugs such as statins, tacrine, and antituberculosis medications, namely INH (Isonicotinic acid hydrazide) [48]. Their continued use often leads to normalization or stabilization of LTs [43].

### 4.6. Intrinsic Liver Injury

EMA also argued that in clinical trials there is a need of early search for intrinsic liver injury and proposed as potential biomarkers total HMGB-1, total and caspase-cleaved keratin-18, miR-122, and GLDH; these parameters should be incorporated within 24 hours in early stage clinical trials before ALT increases (Table 1) [20]. However, some of these parameters are not liver specific [21] or are also suggested to be used for early detection of idiosyncratic DILI (Table 1). Overall, these suggestions are difficult to reconcile, since any potential intrinsic liver injury must have been excluded prior to clinical trials by appropriate measures, otherwise subjects run a high risk of liver injury that could have been prevented (Table 1).

## 5. Idiosyncratic Drug Induced Liver Injury

### 5.1. Pathogenetic Aspects

Developing new biomarkers that would improve the diagnosis or better characterize the clinical course of idiosyncratic DILI requires understanding of pathogenetic events and mechanisms of this liver injury, not or hardly reproducible in experimental studies [1,2,3,4,5,6,7,8,9,10]. Steps from hepatocyte uptake of the drug to cell death are described in various review articles [21,48,49,50,51,52]. For obvious reasons, a direct access to the liver of patients with idiosyncratic DILI is not feasible, except perhaps through invasive liver biopsy. This would allow to get tissue specimens for liver histology or electron microscopy, but such approach just for scientific purposes must clearly be ruled out for ethical reasons. Therefore, assessing mechanisms of idiosyncratic DILI in order to search for potential biomarkers has to analyze parameters in fluids such as blood or urine which could reflect metabolic changes in the liver, focusing for instance on (1) organelle injuries or (2) specific pathological biochemical processes. Potential biomarkers must fulfill at least two criteria, liver specificity and drug specificity, but little evidence exists that these requirements are met. Proposed parameters seemingly reflect in part injuries of other organs or caused by other toxins rather than drugs (Table 1).

Most popular is a three-step working model of idiosyncratic DILI mechanism [49,51]. In the first step, drugs or their metabolites cause direct cell stress, trigger immune reactions and impair mitochondrial functions. This initial hit causes in a second step mitochondrial membrane permeability, which in a third step initiates apoptotic or necrotic cell death [49,51].

As expected, proposals for pathogenetic mechanisms in idiosyncratic DILI involve a large number of intermediates, structural proteins, and liver cell organelles [21,48,49,50,51,52]. Some of them are summarized in Figure 2. Most of the mechanisms were not studied from data of patients with idiosyncratic DILI but with intrinsic DILI due to APAP overdose. Extrapolation of these data to idiosyncratic DILI is highly speculative and not demonstrated. Furthermore, proposals for idiosyncratic DILI in humans [21,48,49,50,51,52] were derived from in vitro experiments or from animal studies using drugs such as acetaminophen that cause intrinsic liver injury.

Considerations on pathogenetic concepts for idiosyncratic DILI were mostly based on circumstantial evidence and remain a matter of debate, in absence studies in humans. For instance, how do we know for sure that drug metabolism in the liver of patients with idiosyncratic DILI leads to reactive metabolites such as ROS (Reactive Oxygen Species), covalent binding, and oxidative stress with hepatic GSH (Glutathione) depletion (Figure 2) and why DILI occurs only in few patients? With respect to the possible immune reactions (Figure 2) [21,48,49,50,51,52], how they were analyzed and how they could induce significant idiosyncratic liver injury in patients who rarely have clinical or laboratory signs of immune reactions? These questions may appear provocative, but are at the heart of the search for biomarkers in idiosyncratic DILI [20,21,22,23,24,25,26,27,28,29,30,31].

### 5.2. Clinical Characteristics

Patients with idiosyncratic DILI present with a broad spectrum of signs and symptoms, which include jaundice similar to other liver diseases (Figure 3) [3]. Excluding alternative causes is mandatory in cases of suspected idiosyncratic DILI, which by definition is a diagnosis of exclusion [3,8,9]. Indeed, an analysis of 13,336 cases of suspected DILI revealed that 34.2% of the cases were not DILI, since the reported liver injury should have been attributed to other causes [9], with a broad range of liver diseases (Table 2) [8,9].

**Figure 4:**
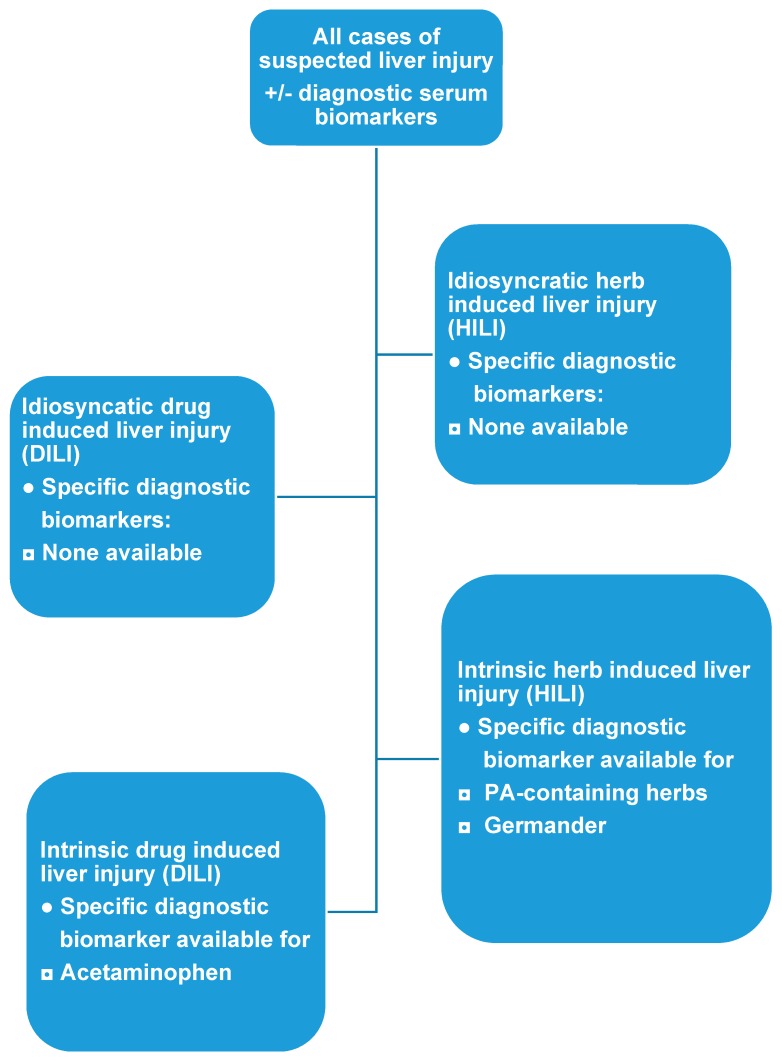
Availability of established specific diagnostic biomarkers for idiosyncratic as compared to intrinsic DILI and HILI. Abbreviations: DILI, Drug induced liver injury; HILI, herb induced liver injury; PAs, Pyrrolizidine alkaloids.

**Figure 5:**
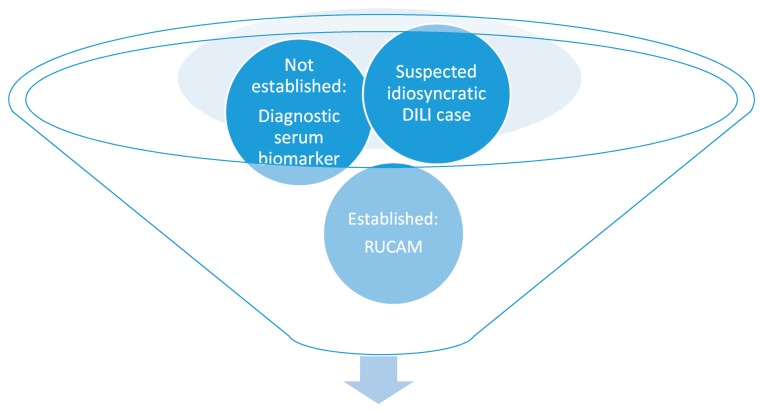
Valid causality assessment of idiosyncratic DILI using the established approach of RUCAM in the absence of a validated diagnostic serum biomarker. Abbreviations: DILI, Drug induced liver injury; RUCAM, Roussel Uclaf Causality Assessment Method.

In clinical practice, the diagnosis of idiosyncratic DILI and HILI cannot rely on specific and sensitive diagnostic biomarker (Table 1, Figure 4) and may only be suspected in patients with increased serum ALT or ALP values under a drug therapy, requiring causality assessment by RUCAM (Figure 5) [3]. Very few drugs cause specific laboratory abnormalities. For instance, specific autoantibodies were reported against cytochrome P450 (CYP) such as CYP2C9 (by tienilic acid, not anymore marketed), CYP1A2 (by dihydralazin), CYP3A4 (by antiepileptic drugs), and CYP2E1 (by halothane) [50]. These assays are still restricted to research laboratories, considering that these autoantibodies may also be seen in drug-exposed persons without concomitant DILI [50]. Similarly, the use of lymphocyte-stimulations tests is problematic due to limited access and lack of standardization and reproducibility in suspected DILI [3,50].

For idiosyncratic DILI, risk factors are known [7,40] such as genetic susceptibility linked to human leucocyte antigen (HLA) alleles [7,53], comedication [7], alcohol use [10,44], preexisting chronic liver diseases [44], drug lipophilicity, extensive metabolic rates and high daily doses of drugs [54,55]. Some factors are scored items in RUCAM, which should preferentially be used to assess overall causality [3].

## 6. RUCAM, Idiosyncratic DILI, and Biomarkers

**Figure 6:**
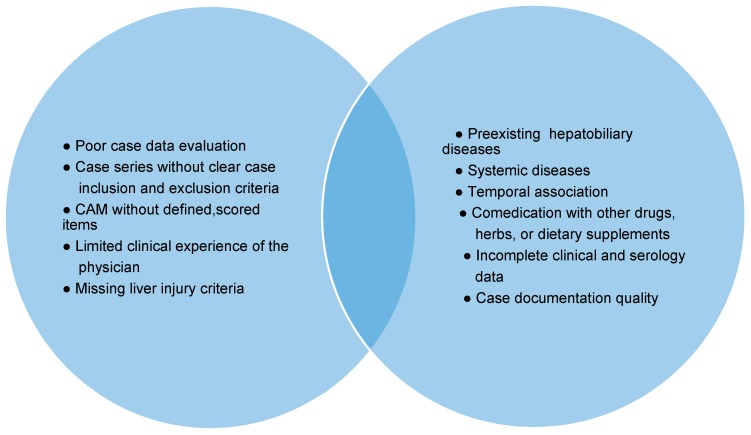
Elements blurring the diagnosis idiosyncratic DILI. Abbreviation: CAM, Causality assessment method.

Idiosyncratic DILI is a complex disease, and causality assessment is complicated by many variables confounding the diagnosis (Figure 6) [9], conditions that cannot be handled appropriately or compensated by potential biomarkers (Table 1) [20,21,22,23,24,25,26,27,28,29,30,31,32] or other approaches including liver histology [56]. RUCAM was set up taking into consideration clinical features, time course of LTs and risk factors, and provides transparent results of causality gradings [1,2,3].

RUCAM is internationally the most frequent tool to assess causality of idiosyncratic DILI [1,2,3,10] and was the first causality assessment method (CAM) that clearly defined criteria of liver injury by using liver test thresholds, based on multiples of ULN (upper limit of normal) of LTs as diagnostic criterion [1]. Liver injury is currently defined by ALT elevation above 5 × ULN [3]. For ALP, elevation above 2 × ULN, provided that ALP is of hepatic origin [3].

In addition, RUCAM was the first CAM that ever recognized the importance of various types of suspected DILI [1]. Based on DILI case analyses and due to the variability of their clinical features, three types of liver injury pattern were defined. The liver injury patterns were classified as hepatocellular, cholestatic, and mixed liver injury [1,3,10]. This classification was later confirmed as discriminant in terms of population and clinical features [57]. Specific and individual scores had to be determined for each liver injury type as they showed differences of clinical features and courses, with focus on challenge, dechallenge, and rechallenge characteristics. For causality assessment using RUCAM, only two instead of three types of liver injury are necessary, i.e. hepatocellular injury and cholestatic/mixed liver injury (Table 3) [1,3,10].

Providing transparency and valid results, RUCAM represents a structured, standardized, quantitative, and liver injury specific diagnostic tool, using scores to quantify final causality levels [3]. Briefly, it attributes scores to key items (Table 3); for instance, the score of search for non-drug causes in RUCAM including markers for acute viral infection, hepatobiliary imaging, cardiac hepatopathy, and autoimmune diseases goes from −3 to +2. The sum of the individual scores provides the final score for each suspected drug. The range of the final scores from +14 to −9 points and allows for grading causality: ≤0, relationship excluded; 1–2, unlikely; 3–5, possible; 6–8, probable; ≥ 9, highly probable [3]. Therefore, RUCAM-based causality grading is objective, does not require expert rounds, has many advantages (Table 4), and differs substantially from other CAMs including expert-based opinion [3]. The latter CAM is, by definition, subjective without scored key items and not transparent [3,11], it may also be challenged due to overdiagnosing and overreporting because data are incomplete or alternative causes and comedication are neglected or minimized [11].

## 7. Intrinsic Drug Induced Liver Injury

### 7.1. Pathogenetic Aspects

The mechanism of intrinsic DILI due to acetaminophen overdose has been well studied, since this drug is best known to cause experimental liver injury as well as severe liver injury including acute liver failure in humans. Many reports focused on this specific type of intrinsic DILI, considering clinical and experimental aspects [7,33,58,59,60,61,62,63,64,65,66,67,68,69,70]. Pathogenetic considerations derived from experimental APAP hepatotoxicity were transferred to idiosyncratic DILI (Table 1, Figure 2) and in support of the proposed three-step events of pathogenetic mechanisms [21,22,23], certainly a risky approach.

Early experimental studies showed that chronic alcohol use increased the risk of APAP liver injury, likely through a mechanism involving in part hepatic microsomal alcohol-induced CYP2E1 [58], as confirmed by subsequent studies [7,59]. To summarize, three phases describe APAP metabolism [59]. The majority of the dose is conjugated by UDP-glucuronosyltransferases (UGT) and sulfotransferase (SULT) to metabolites that are excreted in the urine. The remaining APAP is metabolized mainly by CYP2E1 and to a smaller extent by CYP1A2 and 3A4 to a highly reactive toxic metabolite, *N*-acetyl-para-quinone imine (NAPQI). This metabolite binds preferentially to mitochondrial proteins, especially under conditions of GSH depletion, impairs mitochondrial ATP-synthase, and reduces ATP production. With modified human hepatocytes, the same mechanism of liver injury was shown, starting with GSH depletion and moving through protein adduct and superoxide formation to oxidative stress and mitochondrial membrane dysfunction and disruption, triggering cell necrosis [59]. Consequently, pathogenetic mechanisms are well described for intrinsic DILI due at least to APAP, a single drug, as opposed to idiosyncratic DILI due to a vast variety of drugs.

Of similar interest are pathogenetic aspects of intrinsic HILI as outlined previously [40], especially those related to herbs of the Traditional Chinese Medicine (TCM) containing unsaturated pyrrolizidine alkaloids (PAs) [34,35] or to the herb Germander [36,37,38,39]. However, a more detailed discussion on these aspects is outside the scope of this article.

### 7.2. Clinical Characteristics

Clinical features of intrinsic DILI caused by APAP are well described [33] and similar to those of idiosyncratic DILI [1,4,5,18]. They include abdominal pain, marked weakness, ALT elevation but late jaundice. It is a good example for a RUCAM-based causality assessment in intrinsic DILI [33] used prospectively to ensure data completeness. RUCAM also assessed causality of co-medicated drugs that all had lower causality gradings as compared to APAP [33].

### 7.3. Biomarkers

As opposed to idiosyncratic DILI (Table 1, Figure 4) [20,21,22,23], use of biomarkers is more promising for intrinsic DILI [7]. For instance, valid diagnostic biomarkers were developed on the basis of pathogenetic events leading to APAP liver injury [70] and are in clinical use [7,33,60,61,62,63,64,65,66,67,68,69]. Indeed, acetaminophen-protein adducts in serum and urine are valid biomarkers in patients with acute liver failure without reliable past medical history or other clinical information [70]. In addition, serum parameters such as microRNA-122 or keratin-18 are under consideration to predict the prognosis and outcome of patients with acute liver failure due to APAP [7], but they are certainly not valid diagnostic biomarkers due to a questionable sensitivity and specificity (Table 1) [21].

With serum microsomal epoxide hydrolase, a diagnostic biomarker is available for intrinsic HILI caused by Germander [36,37,38,39,40,50], also known as the herbal TCM Shi Can [40]. Other diagnostic biomarkers such as serum pyrrole-protein adducts are available for HILI due to the herbal TCM San Chi (*Gynura segetum*), which contains unsaturated PAs causing hepatic sinusoidal obstruction syndrome (HSOS) [34,35,40].

## 8. Future Challenges and Perspectives of Biomarkers

**Figure 7:**
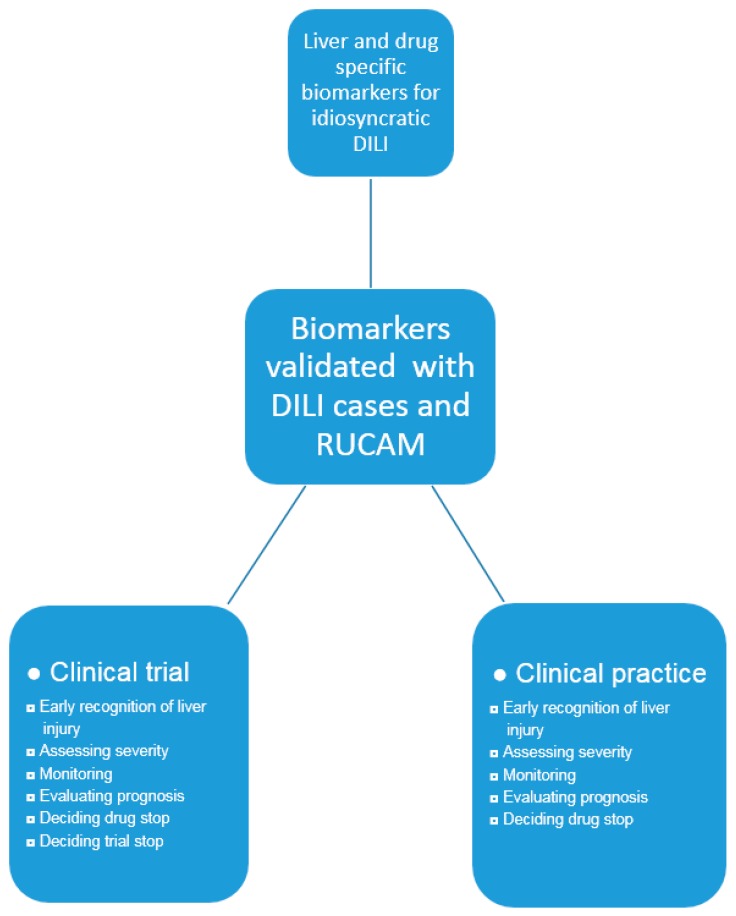
Proposal for biomarker validation and its use in clinical evaluation. Abbreviations: DILI, Drug induced liver injury; RUCAM, Roussel Uclaf Causality Assessment Method.

For diagnostic biomarkers to be used in idiosyncratic DILI, major issues are still to be addressed (Figure 7). In particular, they must be liver specific and drug specific. Validation of new biomarkers must be ascertained with DILI cases of high causality gradings using RUCAM. Developing new diagnostic biomarkers will be difficult in the absence of experimental models that would reflect characteristics of human idiosyncratic DILI. At present, no valid diagnostic biomarker is available or in use which could help establish the diagnosis, would assist or even replace RUCAM for causality assessment in idiosyncratic DILI.

The EMA presented preliminary proposals for new biomarkers in idiosyncratic DILI [20] but the suggestions require refinements (Table 1). A step forward, would be for the EMA to include suggestions made by the FDA [46] in order to achieve a transatlantic common approach for biomarkers. Such cooperation via the SAFE-T (Safer and Faster Evidence-based Translation) Consortium is also recommended [19] and could save financial resources and reduce the need of man-power.

## 9. Conclusions

Efforts are underway in search for a single diagnostic laboratory test to help simplify the diagnosis in patients with suspected liver injury caused by drugs and herbs. No diagnostic biomarker is currently available in routine for DILI diagnosis. DILI is and remains a complex disease, with variable clinical and pathogenetic aspects and a diagnosis of exclusion. RUCAM is the worldwide most applied tool to validly assess causality in suspected idiosyncratic DILI cases. Future studies will have to answer whether new biomarkers can assist RUCAM assessing causality cases of suspected DILI. The validity, specificity and sensitivity of future diagnostic biomarkers must be proven using liver injury cases with established RUCAM-based causality of at least probable but better highly probable causality gradings. Aiming at diagnosing DILI, biomarkers are useless if they merely reflect unspecified liver diseases or toxic liver injury. In addition and ideally, diagnostic biomarkers should be drug or herb specific. To find a single biomarker or a set of biomarkers for an early DILI diagnosis in all patients is a big challenge due to the high number of potential hepatotoxins, the multiple pathogenetic mechanisms, and the variable predisposition of the patients to toxic liver injury.

## Figures and Tables

**Table 1:** Published proposals of possible biomarkers for unspecified liver injury or idiosyncratic DILI in clinical trials.

Proposed Aim	Biomarker	Detection	Comments	References
1. Early recognition of liver injury	CK-18, microRNA-122, total HMGB-1, GLDH, SDH.	Hepatocyte necrosis	Earlier detection unsure as compared to ALT and TBIL. GLDH and SDH are older parameters with unclear advantages.	EMA [20]
			CK-18 fragments not liver specific; marker of caspase cleaved proteins in apoptotic cell death. HMGB-1 necrosis marker, but not liver specific;	Fontana [21]
			microRNA-122 lacks investigations regarding specificity and sensitivity for idiosyncratic DILI.	Teschke et al. [40]
	ccCK-18, M-30	Apoptosis	Presently, unclear advantage.	EMA [20]
			CK-18 fragments not liver specific. M-30 apoptosis marker, no test performance.	Fontana [21]
	M-65	Apoptosis/Necrosis	Total apoptosis and necrosis marker.	Fontana [21]
	microRNA-122, microRNA-192.	Unspecified liver damage	Liver specific release from damaged hepatocytes.	Fontana [21]
	Hyperacetylated HMGB-1, MCSFR1.	Immune activation	Presently, undetermined advantage for any aspect of trial security.	EMA [20]
			Acetylated HMGB-1 innate immune activation factor, acetylation requires mass spectroscopy for detection.	Fontana [21]
2. Outcome	None specified.	NA	Details for DILI outcome not presented, unclear conditions.	EMA [20]
3. Prognosis, progression	Hyperacetylated HMGB-1, Osteopontin, total Keratin-18, MCSFR1.	Risk of progression	For biomarkers of DILI prognosis and monitoring of progression, still yet undetermined advantages as compared to ALT and TBIL in assessing progression to severe DILI (Hy’s law criteria). HMGB-1 is not liver specific.	EMA [20], Fontana [21]
4. Regression	None specified.	NA	Monitoring disease regression by a new biomarker requires more details from EMA, as present LTs are informative enough and need no substitution.	EMA [20]
5. Adaptation	None specified.	NA	A new biomarker for assessing liver adaptation during drug treatment is not justified as available LTs and need no replacement.	EMA [20]
6. Intrinsic liver injury	Total HMGB-1, total Keratin-18, caspase-cleaved keratin-18, microRNA-122, GLDH	Intrinsic liver injury	Exclusion of intrinsic liver injury is a hallmark of pre-clinical studies prior to and not during clinical trials to ensure safety of probands.	EMA [20]
			HMGB-1 is not liver specific.	Fontana [21]
			Cytokeratin-18 fragments are not liver specific.	

Abbreviations: ALT, Alanine aminotransferase; ccCK, caspase-cleaved CytoKeratin; CK, CytoKeratin; DILI, Drug induced liver injury; EMA, European Medicines Agency; GLDH, Glutamate dehydrogenase; HMGB, High Mobility Group Box (protein); Hy, Hyman Zimmerman; LTs, Liver tests; MCSFR, Macrophage colony-stimulating factor receptor; microRNA, microarray RNA; NA, Not available or not assessed; SDH, Sorbitol dehydrogenase; TBIL, Total bilirubin.

**Table 2:** Frequency of specified alternative causes of idiosyncratic DILI.

Alternative Causes	*n*	Frequency %
Biliary diseases	39	11.89
Autoimmune hepatitis	35	10.67
Hepatitis B or C	28	8.54
Hepatic tumor	26	7.93
Ischemic hepatitis	24	7.32
Hepatitis E	20	6.10
Sepsis	20	6.10
Liver injury due to comedication	19	5.79
Viral Hepatitis	18	5.49
Past liver transplantation	17	5.18
Alcoholic liver disease	16	4.88
Fatty liver	9	2.44
Non-alcoholic steatohepatitis	9	2.44
Hepatitis C	6	1.83
Cardiac hepatopathy	5	1.52
Thyroid hepatopathy	4	1.22
Primary biliary cholangitis	3	0.92
Primary sclerosing cholangitis	3	0.92
Gilbert syndrome	3	0.92
CMV Hepatitis	2	0.61
EBV Hepatitis	2	0.61
Hemochromatosis	2	0.61
Wilson disease	2	0.61
Paracetamol overdose	2	0.61
Postictal state	2	0.61
Bone disease	2	0.61
Lymphoma	2	0.61
Preexisting liver cirrhosis	2	0.61
Hepatitis B	1	0.31
Benign recurrent intrahepatic cholestasis	1	0.31
Rhabdomyolysis	1	0.31
Polymyositis	1	0.31
Chlamydial infection	1	0.31
HIV infection	1	0.31
Total	328	100%

Among the study cohort, clearly defined alternative diagnoses were available for 328 patients. Updated details from previous reports [8,9]. Abbreviations: CMV, Cytomegalovirus; DILI, Drug induced liver injury; EBV, Epstein Barr virus; HIV, Human immunodeficiency virus.

**Table 3:** Scores of RUCAM items for hepatocellular injury and cholestatic or mixed liver injury.

Data elements Assessed in RUCAM	Hepatocellular Injury	Cholestatic or Mixed Liver Injury
● Time frame of latency period	From +1 to +2	From +1 to +2
● Time frame of dechallenge	From −2 to +3	From 0 to +2
● Recurrent ALT increase	−2	-
● Recurrent ALP increase	-	0
● Risk factors	0 or +1	
● Separated comedication	From −3 to 0	
● Search for individual alternative causes	From −3 to +2	
● Markers of HAV, HBV, HCV, HEV ● Markers of CMV, EBV, HSV, VZV ● Evaluation of cardiac hepatopathy ● Liver and biliary tract imaging ● Doppler sonography of liver vessels	Requires individual scoring	
● Prior known hepatotoxicity	From 0 to +2	
● Unintentional reexposure	From −2 to +3	

Data above are condensed for a quick overview and adapted from a previous report [3]. Details of each criterion and score are given in the RUCAM worksheet, which in its original, not condensed form is to be used for causality assessment [3]. Abbreviations: ALT, Alanine aminotransferase; ALP, Alkaline phosphatase; CMV, Cytomegalovirus; EBV, Epstein Barr virus; HAV, Hepatitis A virus; HBV, Hepatitis B virus; HCV, Hepatitis C virus; HEV, Hepatitis E virus; HSV, Herpes simplex virus; RUCAM, Roussel Uclaf Causality Assessment Method; VZV, Varicella zoster virus.

**Table 4:** Advantages and limitations of RUCAM.

**Advantages of RUCAM**
Structured and quantitative, liver specific method Prospective use and timely decision Stepwise clinical approach User-friendly and cost-saving method No need of an expert panel Timely use at bedside Clearly defined key items of clinical features and course Full consideration of comedication(s) and alternative causes Consideration is given to prior known hepatotoxicity Incorporation of results of unintentional reexposure Individual scoring system of all key items Validated method (gold standard) Worldwide use □ International registries □ Regulatory agencies □ Pharma companies □ DILI published case reports and case series Transparent documentation Possible, step by step, reevaluation of DILI cases by peers
**Limitations of RUCAM**
RUCAM was not designed for suspected chronic DILI, which is mostly an unrecognized preexisting liver disease RUCAM may require help from expert hepatologists when a suspected DILI occurs on a complicated preexisting liver disease.

Additional details are provided in a recent article [3]. Abbreviations: DILI, Drug induced liver injury; RUCAM, Roussel Uclaf Causality Assessment Method.

## Abbreviations

ALD	Alcoholic liver disease
ALF	Acute liver failure
ALP	Alkaline phosphatase
ALT	Alanine aminotransferase
APAP	*N*-acetyl-para-aminophenol
AST	Aspartate aminotransferase
ATP	Adenosine triphosphate
CMV	Cytomegalovirus.
CAM	Causality assessment method
CK	Cytokeratin
ccCK	Caspase-cleaved CytoKeratin
CYP	Cytochrome P450
Cys	Cysteine
DILI	Drug induced liver injury
EBV	Epstein Barr virus
eDISH	Evaluation of Drug-Induced Serious Hepatotoxicity
EMA	European Medicines Agency
FDA	Food and Drug Administration in Washington, DC
GLDH	Glutamate dehydrogenase
GSH	Glutathione
GST	Glutathione *S*-transferase
HAV	Hepatitis A virus
HBV	Hepatitis B virus
HCV	Hepatitis C virus
HEV	Hepatitis E virus
HILI	Herb induced liver injury
HLA	Human leucocyte antigen
HMGB	High Mobility Group Box (protein)
HSOS	Hepatic sinusoidal obstructions syndrome.
IMI	Innovative Medicines Initiative
INH	Isonicotinic acid hydrazide
LTs	Liver tests
MCSFR-1	Macrophage colony-stimulating factor receptor-1
microRNA	Microarray RNA
NAFLD	Non-alcoholic fatty liver disease
NAPQI	*N*-acetyl-para-quinone imine
NASH	Non-alcoholic steatohepatitis
MCSFR	Macrophage colony stimulating factor receptor
PAs	Pyrrolizidine alkaloids
PCR	Polymerase chain reaction.
R	Ratio
ROS	Reactive oxygen species
RUCAM	Roussel Uclaf Causality Assessment Method
SDH	Sorbitol dehydrogenase
SAFE-T	Safer and Faster Evidence-based Translation
SULT	Sulfotransferase
TBIL	Total bilirubin
TCM	Traditional Chinese Medicine
UGT	UDP-glucuronosyltransferases
ULN	Upper limit of normal (range)
VZV	Varicella zoster virus

## References

[1] Danan G., Bénichou C. (1993). Causality assessment of adverse reactions to drugs—I. A novel method based on the conclusions of international consensus meetings: Application to drug-induced liver injuries. J. Clin. Epidemiol..

[2] Bénichou C., Danan G., Flahault A. (1993). Causality assessment of adverse reactions to drugs—II. An original model for validation of drug causality assessment methods: Case reports with positive rechallenge. J. Clin. Epidemiol..

[3] Danan G., Teschke R. (2016). RUCAM in drug and herb induced liver injury: The update. Int. J. Mol. Sci..

[4] Zhu Y., Niu M., Chen J., Zou Z.S., Ma Z.J., Liu S.H., Wang R.L., He T.T., Song H.B., Wang Z.X. (2016). Comparison between Chinese herbal medicine and Western medicine-induced liver injury of 1985 patients. J. Gastroenterol. Hepatol..

[5] Lu R.J., Zhang Y., Tang F.L., Zheng Z.W., Fan Z.D., Zhu S.M., Qian X.F., Liu N.N. (2016). Clinical characteristics of drug-induced liver injury and related risk factors. Exp. Ther. Med..

[6] Björnsson E.S. (2016). Hepatotoxicity by drugs: The most common implicated agents. Int. J. Mol. Sci..

[7] Sarges P., Steinberg J.M., Lewis J.H. (2016). Drug-induced liver injury: Highlights from a review of the 2015 literature. Drug Saf..

[8] Teschke R., Frenzel C., Wolff A., Eickhoff A., Schulze J. (2014). Drug induced liver injury: Accuracy of diagnosis in published reports. Ann. Hepatol..

[9] Teschke R., Aithal P., Danan G. (2017). Drug induced liver injury: Alternative causes as variables confounding causality. Exp. Opin. Drug Saf..

[10] Teschke R., Danan G., Kang J., Casey D.C. (2018). Causality assessment methods in drug-induced liver injury. Drug-induced Liver Toxicity, Editors: Minjun Chen and Yvonne Will.

[11] Teschke R., Eickhoff A. (2017). Suspected liver injury and the dilemma of causality. Dig. Dis. Sci..

[12] Wang F.S., Fan J.G., Zhang Z., Gao B., Wang H.Y. (2014). The global burden of liver disease: The major impact in China. Hepatology.

[13] Younossi Z.M., Stepanova M., Affendy M., Fang Y., Younossi Y., Mir H., Srishord M. (2011). Changes in the prevalence of the most common causes of chronic liver diseases in the United States from 1988 to 2008. Clin. Gastroenterol. Hepatol..

[14] Scaglione S., Kliethermes S., Cao G., Shoham D., Durazo R., Luke A., Volk M.L. (2015). The epidemiology of cirrhosis in the United States: A population-based study. J. Clin. Gastroenterol..

[15] Bell L.N., Chalasani N. (2009). Epidemiology of idiosyncratic drug-induced liver injury. Semin. Liver Dis..

[16] Sgro C., Clinard F., Ouazir K., Chanay H., Allard C., Guilleminet C., Lenoir C., Lemoine A., Hillon P. (2002). Incidence of drug-induced hepatic injuries: A French population-based study. Hepatology.

[17] De Valle M.B., Av Klinteberg V., Alem N., Olsson R., Björnsson E. (2006). Drug-induced liver injury in a Swedish University hospital out-patient hepatology clinic. Aliment. Pharmacol. Ther..

[18] Björnsson E.S., Bergmann O.M., Björnsson H.K., Kvaran R.B., Olafsson S. (2013). Incidence, presentation and outcomes in patients with drug-induced liver injury in the general population of Iceland. Gastroenterology.

[19] SAFETY-T Consortium: DILI BM Summary Data Package. Release date 30 September 2016. http://www.imi-safe-t.eu/htdocs/biomarker/drug-induced-injury/activities.html.

[20] EMA Letter of Support for Drug-Induced Liver Injury (DILI) Biomarker. EMA/423870/2016, Edited G. Rassi. Last Updated: 30 September 2016. http://www.ema.europa.eu/docs/en_GB/document_library/Other/2016/09/WC500213479.pdf.

[21] Fontana R.J. (2014). Pathogenesis of idiosyncratic drug-induced liver injury and clinical perspectives. Gastroenterology.

[22] Shi Q., Hong H., Senior J., Tong W. (2014). Biomarkers for drug-induced liver injury. Expert Rev. Gastroenterol. Hepatol..

[23] Senior J.R. (2014). New biomarker for drug induced liver injury: Are they really better? what do they diagnose?. Liver Int..

[24] Lewis J.H. (2015). The art and science of diagnosing and managing drug-induced liver injury in 2015 and beyond. Clin. Gastroenterol. Hepatol..

[25] McGill M.R., Jaeschke H. (2015). MicroRNAs as signaling mediators and biomarkers of drug- and chemical-induced liver injury. J. Clin. Med..

[26] Zheng J., Ji C., Lu X., Tong W., Fan X., Gao Y. (2015). Integrated expression profiles of mRNA and microRNA in the liver of Fructus Meliae Toosendan water extract injured mice. Front. Pharmacol..

[27] Enache L.S., Enache E.L., Ramière C., Diaz O., Bancu L., Sin A., André P. (2014). Circulating RNA molecules as biomarkers in liver disease. Int. J. Mol. Sci..

[28] Li L.M., Wang D., Zen K. (2014). Micro RNAs in drug-induced liver injury. Clin. Transl. Hepatol..

[29] Yang X., Salminen W.F., Schnackenberg L.K. (2012). Current and emerging biomarkers of hepatotoxicity. Curr. Biomark. Find..

[30] Lewis P.J.S., Dear J., Platt V., Simpson K.J., Craig D.G.N., Antoine D.J., French N.S., Dhaun N., Webb D.J., Costello E.M. (2011). Circulating microRNAs as potential markers of human drug-induced liver injury. Hepatology.

[31] Thulin P., Nordahl G., Gry M., Yimer G., Aklillu E., Makonnen E., Aderaye G., Lindquist L., Mattsson C.M., Ekblom B. (2014). Keratin-18 and microRNA-122 complement alanine aminotransferase as novel safety biomarkers for drug-induced liver injury in two human cohorts. Liver Int..

[32] Su Y.W., Chen X., Jiang Z.Z., Wang T., Wang C., Zhang Y., Wen J., Xue M., Zhu D., Zhang Y. (2012). A panel of serum microRNAs as specific biomarkers for diagnosis of compound- and herb-induced liver injury in rats. PLoS ONE.

[33] Sabaté M., Ibáñez L., Pérez E., Vidal X., Buti M., Xiol X., Mas A., Guarner C., Forné M. (2011). Paracetamol in therapeutic dosages and acute liver injury: Causality assessment in a prospective case series. BMC Gastroenterol..

[34] Gao H., Li N., Wang J.Y., Zhang S.C., Lin G. (2012). Definitive diagnosis of hepatic sinusoidal obstruction syndrome induced by pyrrolizidine alkaloids. J. Dig. Dis..

[35] Lin G., Wang J.Y., Li N., Li M., Gao H., Ji Y., Zhang F., Wang H., Zhou Y., Ye Y. (2011). Hepatic sinusoidal obstruction syndrome associated with consumption of *Gynura segetum*. J. Hepatol..

[36] Larrey D. (1997). Hepatotoxicity of herbal remedies. J. Hepatol..

[37] Larrey D., Faure S. (2011). Herbal medicine hepatotoxicity: A new step with development of specific biomarkers. J. Hepatol..

[38] Larrey D., Faure S. (2012). Reply to: “Herbal medicine hepatotoxicity revisited”. J. Hepatol..

[39] Larrey D., Vial T., Pauwels A., Castot A., Biour M., David M., Michel H. (1992). Hepatitis after germander (*Teucrium. chamaedrys*) administration: Another instance of herbal medicine hepatotoxicity. Ann. Intern. Med..

[40] Teschke R., Larrey D., Melchart D., Danan G. (2016). Traditional Chinese Medicine (TCM) and herbal hepatotoxicity: RUCAM and the role of novel diagnostic biomarkers such as microRNAs. Medicines.

[41] Teschke R., Eickhoff A. (2015). Herbal hepatotoxicity in traditional and modern medicine: Actual key issues and new encouraging steps. Front. Pharmacol..

[42] Frenzel C., Teschke R. (2016). Herbal hepatotoxicity: Clinical characteristics and listing compilation. Int. J. Mol. Sci..

[43] Teschke R., Danan G. (2016). Diagnosis and management of drug-induced liver injury (DILI) in patients with pre-existing liver disease. Drug Saf..

[44] Teschke R., Danan G. (2017). Drug-induced liver injury: Is chronic liver disease a risk factor and a clinical issue?. Expert Opin. Drug Metab. Toxicol..

[45] Chen E.Y., Baum K., Collins W., Löve A., Merz M., Olafsson S., Björnsson E.S., Lee W.M. (2012). Hepatitis E masquerading as drug-induced liver injury. Hepatology.

[46] FDA Guidance for Industry, Drug-Induced Liver Injury: Premarketing Clinical Evaluation. http://www.fda.gov/downloads/Drugs/GuidanceComplianceRegulatoryInformation/Guidances/UCM174090.pdf.

[47] Senior J.R. (2014). Evolution of the Food and Drug Administration approach to liver safety assessment for new drugs: Current status and challenges. Drug Saf..

[48] Au J.S., Navarro V.J., Rossi S. (2011). Review article: Drug induced liver injury—Its pathophysiology and evolving diagnostic tools. Aliment. Pharmacol. Ther..

[49] Suk K.T., Kim D.J. (2012). Drug-induced liver injury: Present and future. Clin. Mol. Hepatol..

[50] Abboud G., Kaplowitz N. (2007). Drug-induced liver injury. Drug Saf..

[51] Russmann S., Kullak-Ublick G.A., Grattagliano I. (2009). Current concepts of mechanisms in drug-induced hepatotoxicity. Curr. Med. Chem..

[52] Kaplowitz N. (2004). Drug-induced liver injury. Clin. Infect. Dis..

[53] Aithal G.P., Grove J.I. (2015). Genome-wide association studies in drug-induced liver injury: Step change in understanding the pathogenesis. Semin. Liver Dis..

[54] Chen M., Borlak J., Tong W. (2013). High lipophilicity and high daily dose of oral medications are associated with significant risk for drug-induced liver injury. Hepatology.

[55] Chen M., Borlak J., Tong W. (2014). Predicting idiosyncratic drug-induced liver injury—Some recent advances. Expert Rev. Gastroenterol. Hepatol..

[56] Teschke R., Frenzel C. (2014). Drug induced liver injury: Do we still need a routine liver biopsy for diagnosis today?. Ann. Hepatol..

[57] Chalasani N., Bonkovsky H.L., Fontana R., Lee W., Stolz A., Talwalkar J., Reddy K.R., Watkins P.B., Navarro V., Barnhart H. (2015). Features and outcomes of 889 patients with drug-induced liver injury: The DILIN Prospective Study. Gastroenterology.

[58] Teschke R., Stutz G., Strohmeyer G. (1979). Increased paracetamol-induced hepatotoxicity after chronic alcohol consumption. Biochem. Biophys. Res. Commun..

[59] Yoon E., Babar A., Choudhary M., Kutner M., Pyrsopoulos N. (2016). Acetaminophen-induced hepatotoxicity: A comprehensive update. J. Clin. Transl. Hepatol..

[60] Smilkstein M.J., Knapp G.L., Kulig K.W., Rumack B.H. (1988). Efficacy of oral N-acetylcysteine in the treatment of acetaminophen overdose. N. Engl. J. Med..

[61] Mason A., Sallie R. (1995). What causes fulminant hepatic failure of unknown etiology?. Am. J. Clin. Pathol..

[62] Shakil A.O., Kramer D., Mazariegos G.V., Fung J.J., Rakela J. (2000). Acute liver Failure: Clinical features, outcome analysis, and applicability of prognostic criteria. Liver Transpl..

[63] Larson A.M., Polson J., Fontana R.J., Davern T.J., Lalani E., Hynan L.S., Reisch J.S., Schiødt F.V., Ostapowicz G., Shakil A.O. (2005). Acetaminophen-induced acute liver failure: Results of a United States multicentre, prospective study. Hepatology.

[64] Bower W.A., Johns M., Margolis H.S., Williams I.T., Bell B. (2007). Population-based surveillance for acute liver failure. Am. J. Gastroenterol..

[65] Bernal W., Auzinger G., Dhawan A., Wendon J. (2010). Acute liver failure. Lancet.

[66] Reuben A., Koch D.G., Lee W.M., The Acute Liver Failure Study Group (2010). Drug-induced acute liver failure: Results of a U.S. multicenter, prospective study. Hepatology.

[67] Khandelwal N., James L.P., Sanders C., Larson A.M., Lee W.M., The Acute Liver Failure Study Group (2011). Unrecognized acetaminophen toxicity as a cause of indeterminate acute liver failure. Hepatology.

[68] Lee W.M., Larson A.M., Stravitz R.T. AASLD Position Paper: The Management of Acute Liver Failure: Update 2011. https://www.aasld.org/sites/default/files/guideline_documents/alfenhanced.pdf.

[69] Nakayama N., Oketani M., Kawamura Y., Inao M., Nagoshi S., Fujiwara K. (2012). Algorithm to determine the outcome of patients with acute liver failure: A data-mining analysis using decision trees. J. Gastroenterol..

[70] Davern T.J., James L.P., Hinson J.A., Polson J., Larson A.M., Fontana R.J., Lalani E., Munoz S., Shakil A.O., Lee W.M. (2006). Measurement of serum acetaminophen-protein adducts in patients with acute liver failure. Gastroenterology.

